# An Updated Meta-Analysis on Cerebral Embolic Protection in Patients Undergoing Transcatheter Aortic Valve Intervention Stratified by Baseline Surgical Risk and Device Type

**DOI:** 10.1016/j.shj.2023.100178

**Published:** 2023-04-04

**Authors:** Safi U. Khan, Salman Zahid, Mohamad A. Alkhouli, Usman Ali Akbar, Syed Zaid, Hassaan B. Arshad, Stephen H. Little, Michael J. Reardon, Neal S. Kleiman, Sachin S. Goel

**Affiliations:** a Department of Cardiology, Houston Methodist DeBakey Heart & Vascular Center, Houston, Texas, USA; b Sands-Constellation Heart Institute, Rochester General Hospital, Rochester, New York, USA; c Division of Interventional Cardiology, Mayo Clinic, Rochester, Minnesota, USA; d Department of Medicine, North Shore University Hospital, New York, New York, USA; e Department of Cardiovascular Surgery, Houston Methodist DeBakey Heart & Vascular Center, Houston, Texas, USA

**Keywords:** Cerebral embolic protection, Stroke, Transcatheter aortic valve intervention

## Abstract

**Background:**

Transcatheter aortic valve intervention (TAVI) can lead to the embolization of debris. Capturing the debris by cerebral embolic protection (CEP) devices may reduce the risk of stroke. New evidence has allowed us to examine the effects of CEP in patients undergoing TAVI. We aimed to assess the effects of CEP overall and stratified by the device used (SENTINEL or TriGuard) and the surgical risk of the patients.

**Methods:**

We selected randomized controlled trials using electronic databases through September 17, 2022. We estimated random-effects risk ratios (RR) with (95% confidence interval) and calculated absolute risk differences at 30 days across baseline surgical risks derived from the TAVI trials for any stroke (disabling and nondisabling) and all-cause mortality.

**Results:**

Among 6 trials (n = 3921), CEP vs. control did not reduce any stroke [RR: 0.95 (0.50-1.81)], disabling [RR: 0.75 (0.18-3.16)] or nondisabling [RR: 0.99 (0.65-1.49)] strokes, or all-cause mortality [RR: 1.23 (0.55-2.77)]. However, when analyzed by device, SENTINEL reduced disabling stroke [RR: 0.46 (0.22-0.95)], translating into 6 fewer per 1000 in high-risk, 3 fewer per 1000 in intermediate-risk, and 1 fewer per 1000 in low surgical-risk patients. CEP vs. control did not reduce the risk of any bleeding [RR: 1.03 (0.44-2.40)], major vascular complications [RR: 1.41 (0.57-3.48)], or acute kidney injury [RR: 1.36 (0.57-3.28)].

**Conclusions:**

This updated meta-analysis showed that SENTINEL CEP might reduce disabling stroke in patients undergoing TAVI. Patients with high and intermediate surgical risks were most likely to derive benefits.

## Introduction

Transcatheter aortic valve intervention (TAVI) has a procedural risk of stroke (1.2% to 6.7%) within the first few days after the procedure, attributable to the embolization of valve debris.^1^^,^^2^ While studies have reported a higher frequency of cerebral embolic events as assessed using diffusion-weighted magnetic resonance imaging,^3^ clinical strokes are considered more devastating.^4^^,^^5^ In the PARTNER trial, the 30-day stroke rate was 3.3%, associated with increased mortality over a year.^6^ In the STS/TVT (Society of Thoracic Surgeon–Transcatheter Valve Therapy) registry, the 30-day postprocedural stroke rate was 2.3%, resulting in 13% higher 30-day mortality and 43% lower discharge rates to home than those without stroke.^5^

Randomized controlled trials have assessed the utility of cerebral embolic protection (CEP) devices in preventing periprocedural stroke. Most data available are for the Claret SENTINEL (Boston Scientific, US) and TriGuard (Keystone Heart Ltd, Israel) systems. Among TriGuard trials, the DEFLECT III trial (TriGuard HDH) showed safety and resulted in more freedom from ischemic brain lesions, fewer neurological deficits, and improved cognitive function in some domains at discharge and 30 days with CEP.^7^ The REFLECT I (TriGuard HDH)^8^ and REFLECT II (10) (TriGuard 3) demonstrated safety but did not show efficacy in terms of mortality, stroke, or other neurological outcomes.

MISTRAL-C showed that the SENTINEL system tended toward fewer new brain lesions on magnetic resonance imaging than the control for the SENTINEL device.^9^ In the SENTINEL trial, the device captured debris in 99% of patients, demonstrated safety,^10^ and reduced post-TAVI (72 ​hours) stroke by 5.2% in posthoc analysis.^11^ These results led to the approval of the SENTINEL device by the Food and Drug Administration for preventing periprocedural stroke during TAVI. However, since all these trials were small and underpowered to detect differences in clinical stroke, the larger PROTECTED TAVI trial was conducted to investigate the effects of CEP on the risk of periprocedural stroke during TAVI.^12^ Although the trial failed to meet the primary endpoint of all strokes at 72 ​hours or discharge, the 95% confidence intervals (CI) (−1.7% to 0.5%) showed that the benefits of therapy could not be excluded. Furthermore, there was a statistically significant reduction in disabling stroke.

In this context, new evidence allows us to pool individual trial data to assess the role of CEP in protecting against stroke during TAVI, stratified by device type. And since the 30-day stroke and mortality risk varied in the clinical trials comparing TAVI vs. surgical aortic valve intervention (SAVI), corresponding to the baseline surgical risk of the patients,^13^ we also investigated the effects of CEP across surgical risks of patients.

## Methods

We performed this trial-level meta-analysis according to the Cochrane Collaboration guidelines and reported following the Preferred Reporting Items for Systematic Reviews and Meta-Analysis.^14^^,^^15^

### Data Sources, Searches, and Study Selection

We performed a comprehensive literature search without language restriction using Medline, EMBASE, and the CENTRAL databases through September 17, 2022 using broad search terms (“cerebral embolic protection,” “CEP,” “CPD,” “transcatheter aortic valve intervention,”, “TAVI,” “TAVR,” “stroke,” and “mortality”) (Supplemental Table 1).

The prespecified inclusion criteria were: (1) randomized controlled trials comparing CEP devices (SENTINEL or TriGUARD systems) in adult patients with aortic stenosis (AS) undergoing TAVI; and (2) trials must report outcomes of interest. We excluded CLEAN TAVI^16^ and EMBOLI-X^17^ from our main analysis since they used Claret Montage and EMBOL-X devices, respectively. However, these trials were included in the sensitivity analysis to assess whether CEP with any device reduces outcomes. We removed duplicates and screened the remaining articles at the title and abstract level and then at the full-text level (Supplemental Figure 1). Two authors (S.U.K. and S.Z.) independently conducted the study search and selection process and resolved conflicts by discussion and mutual consensus.

### Data Extraction and Risk of Bias Assessment

Two reviewers (S.U.K. and U.A.A.) independently abstracted the data onto the data collection sheets, appraised the data accuracy, performed a risk of bias assessment, and resolved discrepancies by discussing or referring to the original publication. We abstracted the data on the characteristics of trials and participants (age, sex, comorbidities, follow-up duration, valve type, inclusion/exclusion criteria, and definition of primary endpoints) (Supplemental Table 2), the number of events, and sample sizes. In addition, we abstracted data on the intention to treat principles.

We used a Cochrane risk of bias assessment tool for randomized controlled trials.^16^ We assessed the risk of bias at the study level across the following domains: bias due to the randomization process, allocation concealment, blinding of participants and personnel, outcome assessment, incomplete outcome data, and selective reporting (Supplemental Table 3).

### Outcomes of Interest

We primarily focused on any stroke, disabling and nondisabling strokes, and all-cause mortality. Additional endpoints included any bleeding, major vascular complications, and acute kidney injury (AKI). We abstracted the outcomes after 30 days.

### Data Synthesis and Summary Measures

We performed a frequentist pairwise meta-analysis for all patients. First, we measured risk ratios (RRs) with 95% CIs. Then we calculated absolute effects for any stroke (disabling and nondisabling) and all-cause mortality from RRs obtained from meta-analysis and the baseline risk of these outcomes at 30 days from the landmark trials of TAVI vs. SAVI (Supplemental Table 4). To estimate disabling and nondisabling stroke risk, we estimated the proportion of events contributing to any stroke in CEP trials and then applied the proportional event rate to baseline stroke rates abstracted from TAVI trials. We grouped the TAVI vs. SAVI trials according to the Society of Thoracic Surgery-predicted risk of mortality (STS-PROM) score (high, intermediate, and low risk). Finally, we reported the absolute effects of outcomes according to baseline surgical risk for any stroke and all-cause mortality at 30 days (Supplemental Figure 1).

### Statistical Analysis

We pooled outcomes using a random-effects model. We applied the DerSimonian and Laird method for the estimation of τ.^17^ We used *I*^2^ statistics to measure the extent of unexplained statistical heterogeneity: *I ≥* 50% was considered a high degree of between-study statistical heterogeneity (Supplemental Table 5).^18^ We did not assess publication bias since the number of trials were <10. We performed analyses according to the SENTINEL and TriGuard devices. Sensitivity analyses comprised the addition of the CLEAN TAVI and EMBOLI-X trials to assess the overall impact of CEP on summary estimates. For all analyses, we set statistical significance at 5%. We used Comprehensive meta-analysis V 3.0 (Biostat, Englewood, NJ, USA) and MAGICapp (https://magicevidence.org/) for all analyses.

## Results

### Study Search and Trial Characteristics

Our initial electronic search yielded 995 citations; 682 were reviewed after removing duplicates, 143 full-text articles were reviewed after screening at the title and abstract level, and ultimately 6 trials (3921 patients)^7^, ^8^, ^9^, ^10^^,^^12^^,^^19^ were included in the main analysis, and 2 trials (130 patients)^20^^,^^21^ were included in the sensitivity analysis (Supplemental Figure 2; Supplemental Table 6). Three trials (3418 patients)^9^^,^^10^^,^^12^ used SENTINEL CEP, and 3 trials (508 patients)^7^^,^^8^^,^^19^ used the TriGuard system. The median age of participants was 81 (Q1-Q3: 80 to 82) years, and the median proportion of women was 51% (Q1-Q3: 43% to 55%) (Table 1). The outcomes were reported after a follow-up of 30 days. All trials had a low to moderate risk of bias. We did not detect statistical heterogeneity (I^2^ = 0) for the main endpoints.

### Any Stroke and All-Cause Mortality

**Figure 1:**
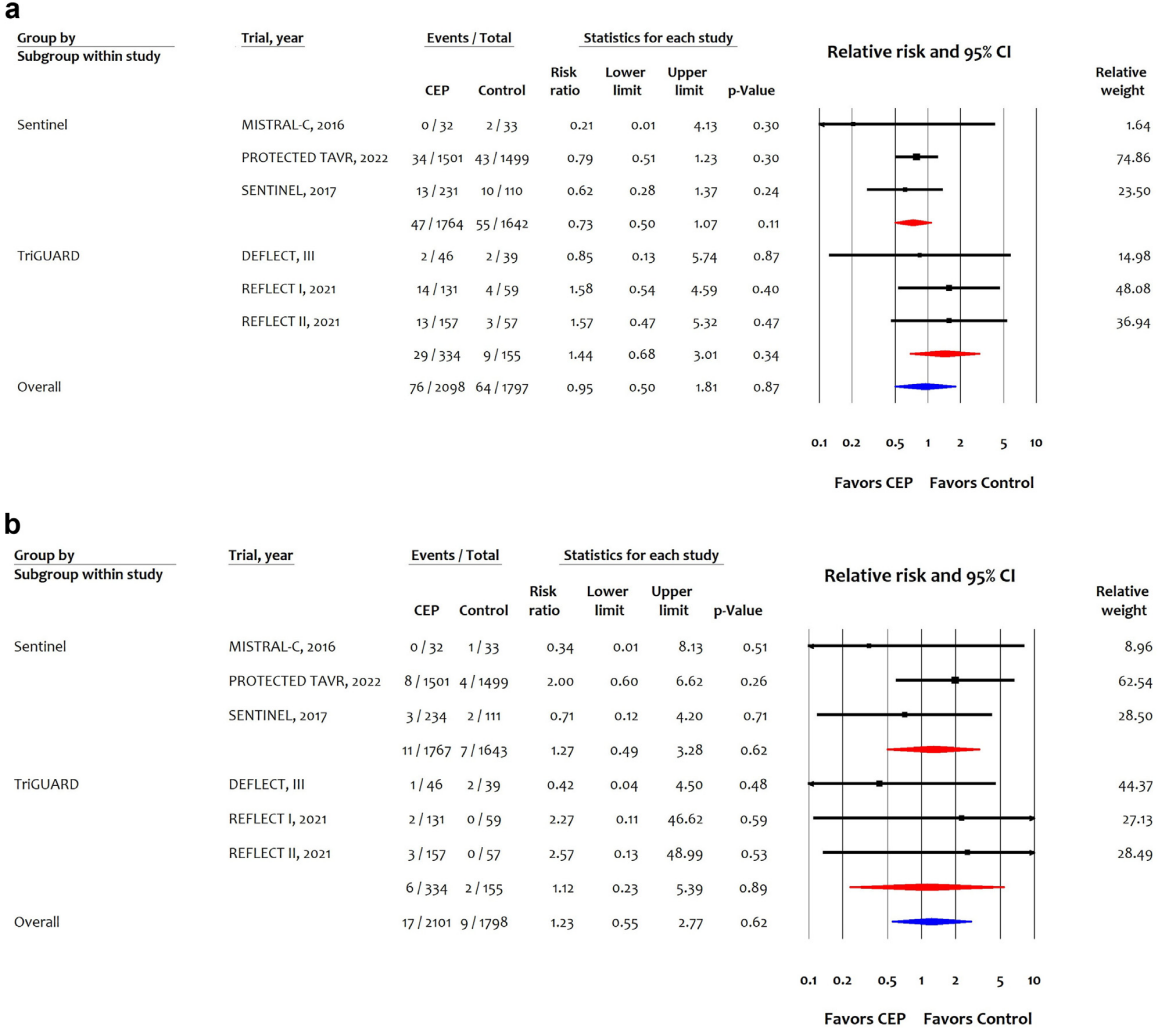
Effects of cerebral embolic protection stratified by device type on (a) any stroke and (b) all-cause mortality. Abbreviations: CEP, cerebral embolic protection; CI, confidence interval; TAVR, transcatheter aortic valve replacement.

Six trials (3895 participants) reported 140 events of any stroke; 3 trials (3406 patients) of SENTINEL reported 102 events; and 3 trials (489 participants) of TriGuard CEP reported 38 events of any stroke. Overall, CEP was not associated with reducing any stroke (RR: 0.95 (95% CI: 0.50-1.81). Both TriGuard (RR: 1.44 (95% CI: 0.68-3.01) and SENTINEL (RR: 0.73 [95% CI: 0.50-1.07; Figure 1A]) showed no significant reduction in any stroke compared with control. In absolute terms, the SENTINEL was associated with 10 fewer (95% CI: 19 fewer to 3 more) per 1000 in high-risk, 6 fewer (95% CI: 11 fewer to 2 more) per 1000 in intermediate-risk and 1 fewer (95% CI: 2 fewer to 0 fewer) per 1000 in low surgical risk at 30 days (Table 2).

**Table 1:** Baseline Demographics of trial populations.

	MISTRAL-C, 2016 (11)	SENTINEL, 2017 (12)	PROTECTED TAVI, 2022 (14)	DEFLECT III, 2015 (8)	REFLECT I, 2021 (9)	REFLECT II, 2021 (10)
CEP device	Claret Sentinel	Claret Sentinel	Claret Sentinel	TriGuard HDH	TriGaurd HDH	TriGUARD 3
TAVI valve	Edwards Sapien 3 (54%), Medtronic CoreValve (25%), Edwards Sapien XT (15%), Balloon dilatation (5%), Portico (1%)	Sapien XT (17.8%), Sapien 3 (52.4%), CoreValve 3.9%, CoreValve Evolut R (25.9%)	Sapien 3 (64%), Evolut R/Evolut PRO (24%), Lotus (0.01%), ACURATE (0.06%), Portico (0.02%)	Edwards Sapien (8.2%), Sapien XT (11.8%), Sapien 3 (43.5%), CoreValve (30.6%), other (3.5%)	CoreVavle (0.01%), CoreValve Evolut R (25%), CoreValve Evolut PRO (0.08%), SAPIEN 3 (58.52%), other (0.03%)	CoreValve (36.9%), Sapien (62.4%), other (0.6%)
STS score, mean	5	6	3	7	5	4
Logistic EuroScore, mean	-	--	5	9	5	3
CEP/control	32/33	234/119	1501/1499	46/39	141/63	157/57
Age, yrs, mean ± SD/median (Q1-Q3)	82 (78-85)	83 (77-87)	79 ± 8	82 ± 6	80 ± 7	80 ± 8
No. of women	31 (48%)	131 (54%)	1197 (40%)	45 (55%)	61 (43%)	121 (56%)
NYHA class, III/IV	21 (32%)	199 (83%)	-	36 (36%)	96 (70%)	114 (53%)
Coronary artery disease	-	127 (40%)	1730 (57%)	10 (10%)	-	-
Prior CABG	-	40 (16%)	-	32 (38%)	29 (21%)	40 (19%)
Prior PCI	-	40 (16%)	1043 (35%)	36 (42%)	34 (26%)	64 (30%)
Hypertension	44(68%)	-	2618 (87%)	65 (76%)	-	-
Atrial fibrillation	16 (28%)	79 (32%)	980 (33%)	24 (28%)	45 (33%)	61 (28%)
Diabetes	13(20%)	82 (34%)	1023 (34%)	19 (22%)	60 (43%)	84 (39%)
Kidney disease	-	-	-	21 (25%)	27 (20%)	53 (25%)
Follow-up, days∗	30	30	30	30	30	30

CEP = cerebral embolic protection device, TAVI = transcatheter aortic valve intervention, CABG = coronary artery bypass graft; CAD = coronary artery disease; NYHA =New York Heart Association; PCI = percutaneous coronary intervention; STS = Society of Thoracic Surgeons; TAVR = transcatheter aortic valve replacement; REFLECT= Randomized Evaluation of TriGuard 3 Cerebral Embolic Protection After Transcatheter, PROTECTED TAVI = Stroke PROTECTion With SENTINEL During transcatheter aortic valve intervention.

∗ 30-day data was abstracted.

**Table 2:** Absolute risk differences for cerebral embolic protection for stroke and all-cause mortality at 30 days

Outcome	Baseline risk∗	Absolute risk difference per 1000 persons (95% confidence interval)		
		Overall (6 trials; 3921 patients)	SENTINEL (3 trials; 3418 patients)	TriGuard (3 trials; 503 patients)
		Cerebral embolic protection vs. control (event per 1000)		
Any stroke		RR: 0.95 (0.50-1.81)	RR: 0.73 (0.50-1.07)	RR: 1.44 (0.68-3.01)
High risk	38 per 1000	2 fewer (19 fewer–31 more)	10 fewer (19 fewer–3 more)	17 more (12 fewer–76 more)
Intermediate risk	22 per 1000	1 fewer (11 fewer–18 more)	6 fewer (11 fewer–2 more)	10 more (7 fewer–44 more)
Low risk	5 per 1000	0 fewer (2 fewer–4 more)	1 fewer (2 fewer–0 fewer)	2 more (7 fewer–10 more)
All-cause mortality		RR: 1.23 (0.55-2.77)	RR: 1.27 (0.49-3.28)	RR: 1.12 (0.23-5.39)
High risk	33 per 1000	8 more (15 fewer–58 more)	9 more (17 fewer–75 more)	4 more (25 fewer–145 more)
Intermediate risk	30 per 1000	7 more (13 fewer–53 more)	8 more (15 fewer–68 more)	4 more (25 fewer–132 more)
Low risk	5 per 1000	1 more (2 fewer–9 more)	1 more (3 fewer–11 more)	1 more (25 fewer–22 more)
Disabling stroke		RR: 0.78 (0.21-2.95)	RR: 0.46 (0.22-0.95)	RR: 1.85 (0.40-8.70)
High risk	11 per 1000	2 fewer (9 fewer–21 more)	6 fewer (9 fewer–1 fewer)	9 more (7 fewer–85 more)
Intermediate risk	6 per 1000	1 fewer (5 fewer–12 more)	3 fewer (5 fewer–1 fewer)	5 more (4 fewer–46 more)
Low risk	1 per 1000	0 fewer (1 fewer–2 more)	1 fewer (2 fewer–0 fewer)	1 more (1 fewer–8 more)
Nondisabling stroke		RR: 0.97 (0.64-1.46)	RR: 0.90 (0.57-1.43)	RR: 1.25 (0.51-3.05)
High risk	27 per 1000	1 fewer (10 fewer–12 more)	3 fewer (12 fewer–12 more)	7 more (13 fewer–55 more)
Intermediate risk	16 per 1000	0 fewer (6 fewer–7 more)	2 fewer (7 fewer–7 more)	4 more (8 fewer–33 more)
Low risk	4 per 1000	0 fewer (1 fewer–2 more)	0 fewer (2 fewer–2 more)	1 more (2 fewer–8 more)

∗ Baseline surgical risk was based on the Society of Thoracic Surgeons (STS) predicted risk of mortality (PROM) score (high: >8%, intermediate: 4% to 8%, and low: <4%) derived from transcatheter aortic valve intervention (TAVI) arm in TAVI vs. surgical aortic valve intervention trials.

Six trials (3899 participants) reported 26 events of all-cause mortality; 3 trials (3410 patients) of SENTINEL reported 18 events, and 3 trials (489 participants) of TriGuard CEP reported 8 events of all-cause mortality. Overall, CEP was not associated with reducing the absolute or relative risk of all-cause mortality (RR: 1.23 [0.55-2.77]); results were consistent for SENTINEL (RR: 1.27 [0.49-3.28] and TriGUARD CEP (RR: 1.12 [0.23-5.39]) (Figure 1B; Table 2).

### Disabling and Nondisabling Stroke

**Figure 2:**
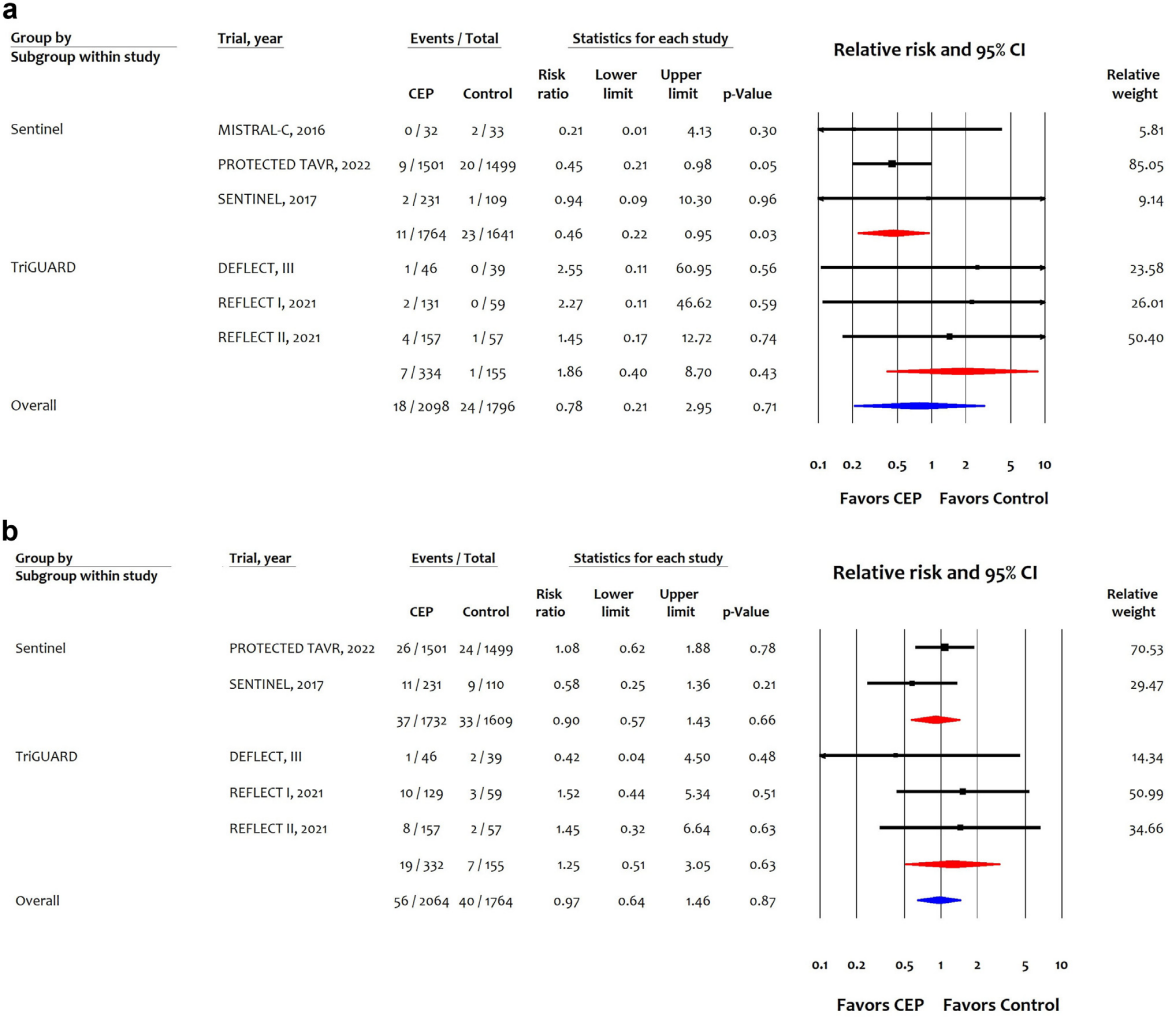
Effects of cerebral embolic protection stratified by device type on (a) disabling stroke and (b) nondisabling stroke. Abbreviations: CEP, cerebral embolic protection; CI, confidence interval; TAVR, transcatheter aortic valve replacement.

Six trials (3894 participants) reported 42 events of disabling stroke; 3 trials (3405 patients) of SENTINEL reported 34 events, and 3 trials (489 participants) of TriGuard CEP reported 8 events of disabling stroke. Overall, CEP was not associated with reducing disabling stroke (RR: 0.78 [95% CI: 0.21-2.95]). TriGuard was not associated with reducing disabling stroke (RR: 1.86 (95% CI: 0.40-8.70), but SENTINEL showed a significant reduction in disabling stroke (RR: 0.46 [95% CI: 0.22-0.95] (Figure 2A). The SENTINEL device was likely associated with 6 fewer events (95% CI: 9 fewer to 1 fewer) of disabling stroke per 1000 patients in high-risk, 3 fewer (95% CI: 5 fewer to 1 fewer) per 1000 patients in intermediate risk, and 1 fewer (2 fewer to 0 fewer) per 1000 patients in low risk (Table 2).

Five trials (3828 participants) reported 96 events of nondisabling stroke, 2 trials (3341 patients) of SENTINEL reported 70 events, and 3 trials (487 participants) of TriGuard CEP reported 26 events. Overall, CEP was not associated with reducing the absolute or relative risk of nondisabling stroke (Figure 2B; Table 2).

### Additional Endpoints and Sensitivity Analysis

**Figure 3:**
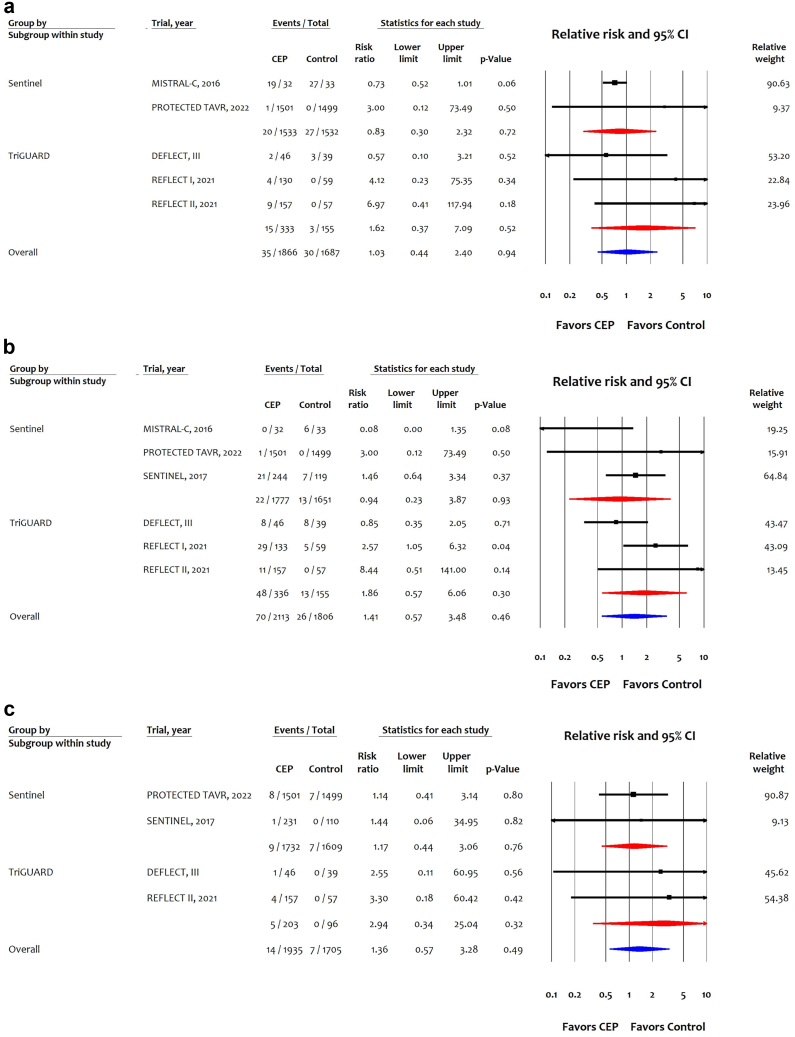
Effects of cerebral embolic protection stratified by device type on (a) major bleeding, (b) major vascular complications and (c) acute kidney injury. Abbreviations: CEP, cerebral embolic protection; CI, confidence interval; TAVR, transcatheter aortic valve replacement.

Compared with control, CEP was not associated with the risk of major bleeding (RR: 1.03 (95% CI: 0.44-2.40); Figure 3A), major vascular complications (RR: 1.41 [95% CI: 0.57-3.48]; Figure 3B), or AKI (RR: 1.36 [0.57-3.28]; Figure 3C). Sensitivity analysis showed consistent results for any stroke (disabling and nondisabling) or all-cause mortality after the addition of the CLEAN TAVI and EMBOLI-X trials (Supplemental Figure 3).

## Discussion

In this updated meta-analysis of randomized controlled trials of more than 3000 patients, CEP did not reduce any stroke or all-cause mortality. However, SENTINEL showed a reduction in disabling stroke. The absolute risk reduction in disabling stroke was proportional to the baseline surgical risk of participants. Patients with high and intermediate surgical risks were most likely to benefit from SENTINEL against disabling stroke, while patients with low surgical risks had an inconsiderable reduction in disabling stroke. Furthermore, CEP was safe and did not increase the risk of major bleeding, vascular complications, or AKI compared with control.

While one may argue that our analysis did not demonstrate a statistically significant difference in any stroke with SENTINEL, overall incident rates were exceedingly low for stroke. Even in the PROTECTED TAVR, both arms showed low incident strokes (CEP 2.3% vs. control: 2.9%).^12^ The trial authors reasoned that treatment patterns have evolved, leading to lower-than-expected stroke rates.^12^ In addition, a lower STS-PROM score (i.e., lower baseline risk), enrollment bias since the CEP device was commercially available in the US only, a residual stroke risk of 2.3% with CEP representing the inability of the CEP to filter all small debris, and that the SENTINEL did not cover the left vertebral artery or protect against hemorrhagic stroke may have contributed to the negative primary outcome of this trial.^12^ Feasibility data on other devices that are intended to provide full coverage to epiaortic arteries, such as the Emblok embolic protection system or the ProtEmbo device, have shown promising results, which may overcome the limitations of filter systems.^22^^,^^23^

In TAVI vs. SAVI trials, 30-day stroke risk varied by patients' surgical risk. In PARTNER 1A, 1B, and US CoreValve, the 30-day risk was 3.8%, 5.0%, and 3.9% during TAVI, respectively. In comparison, this risk was substantially lower in TAVI trials of low-risk patients.^13^ In NOTION, PARTNER 3, and Evolut low risk, the 30-day stroke rates were 1.4, 0%, and 0.5%, respectively. Therefore, it is conceivable that patients with higher baseline risk would yield maximum therapy benefits over those with lower risks. The PROTECTED TAVR included patients at all levels of surgical risks, with an average STS score of 3.3% in the CEP arm and 3.4% in the control arm. While subgroup analyses of PROTECTED TAVR could not specify populations in whom CEP may have potential benefit, there was a signal towards absolute reduction in disabling stroke with CEP in patients with higher STS scores.^12^ Nevertheless, the findings of the ongoing BHF PROTECT-TAVI trial (ISRCTN Registry number: ISRCTN16665769), with a projected enrollment of 7000 patients may have sufficient statistical power to strengthen the existing data and provide further insights into the efficacy of the CEP.

We compared our results with prior meta-analyses. An early meta-analysis on CEP use in TAVI reported no differences in 30-day stroke with CEP.^24^ While Testa and colleagues^25^ showed a reduction in 30-day stroke with CEP, their findings were influenced by a large retrospective study. Similar findings were reported from the TVT registry and administrative datasets for the SENTINEL device,^26^^,^^27^ demonstrating a reduction of stroke. The prior meta-analyses included multiple devices that are not used in routine practice and did not investigate the absolute risks of CEP based on the underlying risk of the included patients. Our updated meta-analysis is unique in that we focused only on randomized controlled trials, contemporary CEP devices, and estimated absolute risk differences to identify patients likely to gain maximum benefits from CEP.^24^ In addition, we performed sensitivity analyses to include trials^20^^,^^21^ of other devices to avoid selection bias.

This meta-analysis has certain limitations. Firstly, this is a study-level meta-analysis, and due to a lack of patient-level characteristics, we could not perform additional subgroup analyses based on age, sex, TAVI access, comorbidities, TAVI valves, and stroke type (ischemic vs. hemorrhagic). Secondly, the included studies were heterogeneous, with different types and generations of CEP devices and types of valves (balloon-expandable vs. self-expandable). Thirdly, variability in methods assessing stroke may have led to an under or overdetection of strokes because many studies did not perform neurologic assessments or event adjudication by experienced neurologists. Finally, our results were mainly driven by the large PROTECTED TAVR trial of the SENTINEL device,^12^ whereas other trials were small with low event rates and imprecise CIs.

In conclusion, our updated meta-analysis showed that SENTINEL CEP might protect against a disabling stroke during TAVI. Furthermore, the SENTINEL CEP was safe and can be offered to select patients with high to intermediate surgical risk. A personalized approach involving shared decision-making between patients and physicians based on patients' baseline risk may result in a meaningful reduction in stroke with the SENTINEL CEP.

## Ethics Statement

The research reported has adhered to the relevant ethical guidelines.

## Funding

The authors have no funding to report.

## Disclosure Statement

S. S. Goel: consultant—Medtronic and speakers’ bureau—Abbott Structural Heart. M. J. Reardon: consultant—Medtronic, Boston Scientific, and Gore Medical. The other authors had no conflicts to declare.
