# Supplementary material for: An Updated Meta-Analysis on Cerebral Embolic Protection in Patients Undergoing Transcatheter Aortic Valve Intervention Stratified by Baseline Surgical Risk and Device Type
Source: Struct Heart. 2023 Apr 4;7(4):100178. doi: 10.1016/j.shj.2023.100178 (PMC10382981; doi:10.1016/j.shj.2023.100178)
Supplement: Supplemental Tables 1-6 and Figures 1-3 [file mmc1.docx]

**Appendix**

**Online Table 1.** Search Strategy (2010-2022)

| Search number | Query | Sort By | Filters | Search Details | | Results |
| --- | --- | --- | --- | --- | --- | --- |
| 14 | Similar articles for PMID: 29728369 | | Clinical Study, Meta-Analysis, Observational Study, Randomized Controlled Trial | | 29728369,28411259,30218830,27765306,29428159,28917515,31091551,31175048,32273098,30467892,27121306,26768742,28895285,25868876,27400304,30154060,31195822,27532914,25341709,29976363,25455466,29550047,28658982,27577965,29221741,31567113,22580248,32518532,24550141,22403768,21087748,33164763,28145039,28797431,27436602,21087749,30676494,30590554,28836953,28029207,27765307,25596724,28566290,28527771,29444998,28521921,30299408,27301396,27601429,29068137,29128473,27637289,26646556,29316204,27930358,28698290,29253108,29301640,29302939,26364995,30341729,29628466,28676065,29759854,27645761,21087747,29861110,27296202,29137967,23652860,27815101,28183467,28289067,28941127,28073567,28104211,27515377,29348010,28830749,28645810,28797420,31542908,29105984,29174824,27890512,29388308,29447771,29929639,29235436,30661140,28183466,27313280,27640028,27412897,27116975,28787505,28648116,29413244,27832845,30354588,28470996,31904829,30019825,28684441,27989061,31184741,29929637,29741978,31705385,31780421,32091552,32091530,30505542,31416047,31680032,30855028,29659154,32273099,32593597,32356620,30406115,32651654,32152782,33258572,32091398,29588728,30858888,30602014,32710845,32171719,29588741,32819800,31864841,32081837,32601823,33417879,32238792[UID] | 43 |
| 13 | ("Transcatheter Aortic Valve Replacement"[Mesh] OR "Heart Valve Prosthesis Implantation"[Mesh]) AND ("CPD"[All Fields] OR "Cerebral"[All Fields]) | | Clinical Study, Meta-Analysis, Observational Study, Randomized Controlled Trial | | ("Transcatheter Aortic Valve Replacement"[MeSH Terms] OR "Heart Valve Prosthesis Implantation"[MeSH Terms]) AND ("CPD"[All Fields] OR "Cerebral"[All Fields]) | 89 |
| 12 | ("Transcatheter Aortic Valve Replacement"[Mesh] OR "Heart Valve Prosthesis Implantation"[Mesh]) AND ("CPD"[All Fields] OR "Cerebral Protection Device"[All Fields]) | | Clinical Study, Meta-Analysis, Observational Study, Randomized Controlled Trial | | ("Transcatheter Aortic Valve Replacement"[MeSH Terms] OR "Heart Valve Prosthesis Implantation"[MeSH Terms]) AND ("CPD"[All Fields] OR "Cerebral Protection Device"[All Fields]) | 4 |
| 11 | ("Transcatheter Aortic Valve Replacement"[Mesh] OR "Heart Valve Prosthesis Implantation"[Mesh]) AND ("CPD"[title/abstract] OR "Cerebral Protection Device"[title/abstract]) | | Clinical Study, Meta-Analysis, Observational Study, Randomized Controlled Trial | | ("Transcatheter Aortic Valve Replacement"[MeSH Terms] OR "Heart Valve Prosthesis Implantation"[MeSH Terms]) AND ("CPD"[Title/Abstract] OR "Cerebral Protection Device"[Title/Abstract]) | 4 |
| 10 | ("Transcatheter Aortic Valve Replacement"[Majr] OR "Heart Valve Prosthesis Implantation"[Majr]) AND ("CPD"[title/abstract] OR "Cerebral Protection Device"[title/abstract]) | | Clinical Study, Meta-Analysis, Observational Study, Randomized Controlled Trial | | ("Transcatheter Aortic Valve Replacement"[MeSH Major Topic] OR "Heart Valve Prosthesis Implantation"[MeSH Major Topic]) AND ("CPD"[Title/Abstract] OR "Cerebral Protection Device"[Title/Abstract]) | 4 |
| 9 | ("Transcatheter Aortic Valve Replacement"[Majr] OR "Heart Valve Prosthesis Implantation"[Majr]) AND ("CPD"[title/abstract] OR "Cerebral Protection Device"[title/abstract]) | | Clinical Study, Meta-Analysis, Randomized Controlled Trial | | ("Transcatheter Aortic Valve Replacement"[MeSH Major Topic] OR "Heart Valve Prosthesis Implantation"[MeSH Major Topic]) AND ("CPD"[Title/Abstract] OR "Cerebral Protection Device"[Title/Abstract]) | 4 |
| 8 | ("Transcatheter Aortic Valve Replacement"[Majr] OR "Heart Valve Prosthesis Implantation"[Majr]) AND ("CPD"[title/abstract] OR "Cerebral Protection Device"[title/abstract]) | | Clinical Study, Meta-Analysis | | ("Transcatheter Aortic Valve Replacement"[MeSH Major Topic] OR "Heart Valve Prosthesis Implantation"[MeSH Major Topic]) AND ("CPD"[Title/Abstract] OR "Cerebral Protection Device"[Title/Abstract]) | 4 |
| 7 | ("Transcatheter Aortic Valve Replacement"[Majr] OR "Heart Valve Prosthesis Implantation"[Majr]) AND ("CPD"[title/abstract] OR "Cerebral Protection Device"[title/abstract]) | | Meta-Analysis | | ("Transcatheter Aortic Valve Replacement"[MeSH Major Topic] OR "Heart Valve Prosthesis Implantation"[MeSH Major Topic]) AND ("CPD"[Title/Abstract] OR "Cerebral Protection Device"[Title/Abstract]) | 2 |
| 6 | ("Transcatheter Aortic Valve Replacement"[Majr] OR "Heart Valve Prosthesis Implantation"[Majr]) AND ("CPD"[title/abstract] OR "Cerebral Protection Device"[title/abstract]) | | Randomized Controlled Trial | | ("Transcatheter Aortic Valve Replacement"[MeSH Major Topic] OR "Heart Valve Prosthesis Implantation"[MeSH Major Topic]) AND ("CPD"[Title/Abstract] OR "Cerebral Protection Device"[Title/Abstract]) | 1 |
| 5 | ("Transcatheter Aortic Valve Replacement"[Majr] OR "Heart Valve Prosthesis Implantation"[Majr]) AND ("CPD"[title/abstract] OR "Cerebral Protection Device"[title/abstract]) | | Most Recent | | ("Transcatheter Aortic Valve Replacement"[MeSH Major Topic] OR "Heart Valve Prosthesis Implantation"[MeSH Major Topic]) AND ("CPD"[Title/Abstract] OR "Cerebral Protection Device"[Title/Abstract]) | 10 |
| 4 | Cerebral protection device, Transcatheter aortic valve replacement | | Clinical Study, Meta-Analysis, Observational Study, Randomized Controlled Trial | | ("cerebrally"[All Fields] OR "cerebrum"[MeSH Terms] OR "cerebrum"[All Fields] OR "cerebral"[All Fields] OR "brain"[MeSH Terms] OR "brain"[All Fields]) AND ("protect"[All Fields] OR "protected"[All Fields] OR "protecting"[All Fields] OR "protection"[All Fields] OR "protections"[All Fields] OR "protective agents"[Pharmacological Action] OR "protective agents"[MeSH Terms] OR ("protective"[All Fields] AND "agents"[All Fields]) OR "protective agents"[All Fields] OR "protectant"[All Fields] OR "protectants"[All Fields] OR "protective"[All Fields] OR "protectively"[All Fields] OR "protectiveness"[All Fields] OR "protectives"[All Fields] OR "protects"[All Fields]) AND ("device s"[All Fields] OR "equipment and supplies"[MeSH Terms] OR ("equipment"[All Fields] AND "supplies"[All Fields]) OR "equipment and supplies"[All Fields] OR "device"[All Fields] OR "instrumentation"[MeSH Subheading] OR "instrumentation"[All Fields] OR "devices"[All Fields]) AND ("transcatheter aortic valve replacement"[MeSH Terms] OR ("transcatheter"[All Fields] AND "aortic"[All Fields] AND "valve"[All Fields] AND "replacement"[All Fields]) OR "transcatheter aortic valve replacement"[All Fields]) | 20 |
| 3 | Cerebral protection device, Transcatheter aortic valve replacement | | Meta-Analysis, Observational Study, Randomized Controlled Trial | | ("cerebrally"[All Fields] OR "cerebrum"[MeSH Terms] OR "cerebrum"[All Fields] OR "cerebral"[All Fields] OR "brain"[MeSH Terms] OR "brain"[All Fields]) AND ("protect"[All Fields] OR "protected"[All Fields] OR "protecting"[All Fields] OR "protection"[All Fields] OR "protections"[All Fields] OR "protective agents"[Pharmacological Action] OR "protective agents"[MeSH Terms] OR ("protective"[All Fields] AND "agents"[All Fields]) OR "protective agents"[All Fields] OR "protectant"[All Fields] OR "protectants"[All Fields] OR "protective"[All Fields] OR "protectively"[All Fields] OR "protectiveness"[All Fields] OR "protectives"[All Fields] OR "protects"[All Fields]) AND ("device s"[All Fields] OR "equipment and supplies"[MeSH Terms] OR ("equipment"[All Fields] AND "supplies"[All Fields]) OR "equipment and supplies"[All Fields] OR "device"[All Fields] OR "instrumentation"[MeSH Subheading] OR "instrumentation"[All Fields] OR "devices"[All Fields]) AND ("transcatheter aortic valve replacement"[MeSH Terms] OR ("transcatheter"[All Fields] AND "aortic"[All Fields] AND "valve"[All Fields] AND "replacement"[All Fields]) OR "transcatheter aortic valve replacement"[All Fields]) | 15 |
| 2 | Cerebral protection device, Transcatheter aortic valve replacement | | Observational Study, Randomized Controlled Trial | | ("cerebrally"[All Fields] OR "cerebrum"[MeSH Terms] OR "cerebrum"[All Fields] OR "cerebral"[All Fields] OR "brain"[MeSH Terms] OR "brain"[All Fields]) AND ("protect"[All Fields] OR "protected"[All Fields] OR "protecting"[All Fields] OR "protection"[All Fields] OR "protections"[All Fields] OR "protective agents"[Pharmacological Action] OR "protective agents"[MeSH Terms] OR ("protective"[All Fields] AND "agents"[All Fields]) OR "protective agents"[All Fields] OR "protectant"[All Fields] OR "protectants"[All Fields] OR "protective"[All Fields] OR "protectively"[All Fields] OR "protectiveness"[All Fields] OR "protectives"[All Fields] OR "protects"[All Fields]) AND ("device s"[All Fields] OR "equipment and supplies"[MeSH Terms] OR ("equipment"[All Fields] AND "supplies"[All Fields]) OR "equipment and supplies"[All Fields] OR "device"[All Fields] OR "instrumentation"[MeSH Subheading] OR "instrumentation"[All Fields] OR "devices"[All Fields]) AND ("transcatheter aortic valve replacement"[MeSH Terms] OR ("transcatheter"[All Fields] AND "aortic"[All Fields] AND "valve"[All Fields] AND "replacement"[All Fields]) OR "transcatheter aortic valve replacement"[All Fields]) | 7 |
| 1 | Cerebral protection device, Transcatheter aortic valve replacement | | Observational Study | | ("cerebrally"[All Fields] OR "cerebrum"[MeSH Terms] OR "cerebrum"[All Fields] OR "cerebral"[All Fields] OR "brain"[MeSH Terms] OR "brain"[All Fields]) AND ("protect"[All Fields] OR "protected"[All Fields] OR "protecting"[All Fields] OR "protection"[All Fields] OR "protections"[All Fields] OR "protective agents"[Pharmacological Action] OR "protective agents"[MeSH Terms] OR ("protective"[All Fields] AND "agents"[All Fields]) OR "protective agents"[All Fields] OR "protectant"[All Fields] OR "protectants"[All Fields] OR "protective"[All Fields] OR "protectively"[All Fields] OR "protectiveness"[All Fields] OR "protectives"[All Fields] OR "protects"[All Fields]) AND ("device s"[All Fields] OR "equipment and supplies"[MeSH Terms] OR ("equipment"[All Fields] AND "supplies"[All Fields]) OR "equipment and supplies"[All Fields] OR "device"[All Fields] OR "instrumentation"[MeSH Subheading] OR "instrumentation"[All Fields] OR "devices"[All Fields]) AND ("transcatheter aortic valve replacement"[MeSH Terms] OR ("transcatheter"[All Fields] AND "aortic"[All Fields] AND "valve"[All Fields] AND "replacement"[All Fields]) OR "transcatheter aortic valve replacement"[All Fields]) | 2 |

**Online Table 2**. Patient selection criteria, outcome, and definition of endpoints.

|  |  |  |  | **Definition** | | |
| --- | --- | --- | --- | --- | --- | --- |
| **Trials, year** | **Inclusion criteria** | **Exclusion criteria** | **Primary outcome** | **Stroke** | **mortality** | **Vascular complications** |
| MISTRAL-C, 2016 | Symptomatic severe AS planned for transfemoral TAVI | Presence of  a permanent pacemaker or automated internal cardiac defibrillator  (AICD) at baseline, a history of prior stroke with sequelae and  dementia. | The numerical difference in new  positive post-procedure diffusion-weighted MRI brain lesions at 2 days after TAVI in potentially protected territories. | VARC-2 | VARC-2 | VARC-2 |
| SENTINEL, 2017 | Severe  symptomatic AS and planned TAVI who were at high surgical risk | Contraindications for right radial or brachial artery | **Primary safety:**  Occurrence of major adverse cardiac and cerebrovascular  events (MACCE) at 30 days compared  with a historical performance goal  **Primary efficacy:**  Reduction in  median total new lesion volume in protected territories  between the device and control arms, as  assessed by diffusion-weighted MRI at 2 to 7 days after  TAVI | VARC-2 | VARC-2 | VARC-2 |
| PROTECTED TAVR, 2022 | Severe symptomatic AS referred for TAVI | Subject has arterial stenosis >70% in either the left common carotid artery or the brachiocephalic artery. • Subject’s brachiocephalic or left carotid artery reveals significant stenosis, ectasia, dissection, or aneurysm at the aortic ostium or within 3 cm of the aortic ostium. • Subject has compromised blood flow to the right upper extremity. • Subject has access vessels with excessive tortuosity. • Subject has uncorrected bleeding disorders. • Subject is contraindicated for anticoagulant and antiplatelet therapy. | All stroke (hemorrhagic, ischemic, or undetermined status) through 72 hours after TAVR procedure or discharge (whichever comes first) as adjudicated by an independent Clinical Events Committee (CEC) and using Neurologic Academic Research Consortium (NeuroARC) definitions | Research Consortium(NeuroARC) | VARC-2 | VARC-2 |
| DEFLECT III, 2015 | Severe symptomatic AS referred for TAVI | Recent myocardial infarction, prior stroke, cardiogenic shock,  contraindications to antiplatelet or anticoagulant therapy, heavily calcified or severely atheromatous aortic arch or  aortic arch anatomy that could prevent positioning and stability of the  device, had contraindications to cerebral MRI | In-hospital MACCE (composed of all cause death, stroke, life threatening bleeding, AKI (Stage 2/3), Coronary obstruction with intervention, major vascular complications and valve related dysfunction) | VARC-2 | VARC-2 | VARC-2 |
| REFLECT I, 2021 | Severe symptomatic AS referred for TAVI | Recent (<72 h) acute myocardial infarction, recent (<6 months) stroke or transient ischaemic attack, cardiogenic shock, impaired renal function (glomerular filtration rate <30 mL/min/1.73 m2), history of bleeding diathesis or coagulopathy or contraindications to antiplatelet or anticoagulant therapy, and prior prosthetic valve implantation (including planned aortic valve-in-valve procedure) | **Primary Safety:**  Composite of all-cause death, stroke, life-threatening or disabling bleeding, stage 2–3 Acute kidney injury, coronary artery obstruction requiring intervention, major vascular complications, and valve-related dysfunction requiring repeat procedure  **Primary Efficacy:**  Composite of (i) all-cause mortality or any stroke at 30 days, (ii) NIHSS worsening from baseline to 2–5 days post-procedure or MoCA worsening (decrease of three points or more from baseline) at 30 days, and (iii) total volume of cerebral ischaemic lesions detected by DW-MRI performed 2–5 days post-procedure | VARC-2 | VARC-2 | VARC-2 |
| REFLECT II, 2021 | Severe AS stenosis with planned transfemoral TAVI | Prior AVR  Stroke/TIA < 6 months  Contraindication to antiplatelet or anticoagulation treatment  eGFR <30 ml/min  CT angiograms of the chest, abdomen, and pelvis were analyzed by the independent CT core lab and reviewed by a screening committee  Severe peripheral vascular disease (iliofemoral MLD <3.5mm)  Severely calcified or atheromatous aorta  Contraindication to MRI | All-cause mortality, stroke, life-threatening or disabling bleeding, stage 2/3 AKI, coronary artery obstruction requiring intervention, major vascular complication, and valve-related dysfunction requiring intervention (VARC 2 defined) | VARC-2 | VARC-2 | VARC-2 |

**Online Table 3.** Risk of bias in the included trials as assessed by the Cochrane risk of bias assessment scale

|  |  |  | **Blinding** | |  |  |  |
| --- | --- | --- | --- | --- | --- | --- | --- |
| **Studies** | Random sequence generation | Allocation concealment | Participants and personnel | Outcome assessment | Incomplete outcome data | Selective reporting | Overall quality |
| MISTRAL-C, 2016 | Unclear | Low risk | Low risk | Low risk | Low risk | Low risk | High |
| SENTIL, 2017 | Low risk | Low risk | Low risk | Low risk | High risk | Low risk | High |
| PROTECTED TAVR, 2022 | Low risk | Low risk | Low risk | Low risk | Low risk | Low risk | High |
| DEFLECT III, 2015 | Unclear | Low risk | Low risk | Low risk | Low risk | Low risk | High |
| REFLECT I, 2021 | Unclear | Unclear | Low risk | Low risk | High risk | Low risk | Moderate |
| REFLECT II, 2021 | Unclear | Unclear | Low risk | Low risk | High risk | Low risk | Moderate |
| *Trials included in sensitivity analysis* |  |  |  |  |  |  |  |
| EMBOL-X, 2015 | Unclear | Unclear | Low risk | Low risk | Low risk | Low risk | Moderate |
| CLEAN TAVI, 2016 | Low risk | Low risk | Low risk | Low risk | Low risk | Low risk | High |

We rated trials as at (i) low risk of bias (green), (ii) some concerns — probably low risk of bias (yellow), (iii) some concerns — probably high risk of bias (orange), or (iv) high risk of bias (red). We rated trials at high risk of bias overall if one or more domains were rated as probably high risk of bias or as high risk of bias; low risk of bias if all domains were rated as probably low risk of bias or low risk of bias.

**Online Table 4.** Baseline risk for stroke and all-cause mortality at 30 days from transcatheter vs. surgical aortic valve intervention trials.

| **Trials** | **Stroke** | **All-cause mortality** |
| --- | --- | --- |
| *High or prohibitive risk* | 3.8 | 3.4 |
| PARTNER 1A | 5.0 | 5.0 |
| PARTNER 1B | 3.9 | 3.3 |
| US CoreValve |  |  |
| *Intermediate surgical risk* |  |  |
| PARTNER 2 | 3.2 | 3.9 |
| SURTAVI | 1.2 | 2.2 |
| *Low surgical risk* |  |  |
| NOTION | 1.4 | 2.1 |
| PARTNER 3 | 0 | 0.4 |
| Evolut Low risk | 0.5 | 0.5 |

We took median of event rate of all trials in each surgical risk to estimate baseline risk for stroke and all-cause mortality

**Online Table 5.** Heterogeneity assessment.

| **Outcome** | **Tau** | **I^2^** |
| --- | --- | --- |
| Any stroke | 0 | 0% |
| Disabling stroke | 0 | 0% |
| Non disabling stroke | 0 | 0% |
| All-cause mortality | 0 | 0% |
| Any or major bleeding | 0 | 0% |
| Major vascular complications | 0.3 | 42% |
| Acute kidney injury | 0 | 0% |

**Online Table 6.** Baseline characteristics of trials and patients included in sensitivity analysis

|  | **EMBOLI-X, 2015** | **Clean TAVI, 2016** |
| --- | --- | --- |
| CPD device | EMBOL-X | Claret |
| TAVI valve | Sapien XT | CoreVavle |
| STS score, mean (SD) | 11.4 (6.9) | 5.6 (3.2) |
| Logistic EuroScore, mean (SD) | 39.2 (13.0) | 16.4% (10.0)* |
| CPD/Control | 14/16 | 50/50 |
| Age, yrs, mean (SD)/median (Q1-Q3) | 81.0 (5.0) | 80 (5.1) |
| No. of women | 10 (71.4%) | 29 (58%) |
| NYHA class, III/IV | - | 16 (32%) |
| Coronary artery disease | - | 26 (52%) |
| Prior CABG | - | 8 (16%) |
| Prior PCI | - | 5 (10%) |
| Hypertension | - | 44 (88%) |
| Atrial fibrillation | - | 17 (34%) |
| Diabetes | - | 20 (40%) |
| Kidney disease | 6 (42.8%) | 21 (43%) |
| *Follow-up, days | 7 | 30 |

*Logistic EuroScore predicts risk of operative mortality with higher accuracy as compared with the standard EuroScore (<10%, low risk; ≥10%-≤20%, intermediate risk; and >20%, high risk), CPD: Cerebral Protection device, TAVI= Transcatheter Aortic Valve Implantation, CABG =coronary artery bypass graft; CAD= coronary artery disease; NYHA =New York Heart Association; PCI =percutaneous coronary intervention; STS = Society of Thoracic Surgeons; TAVR= transcatheter aortic valve replacement.

**Online Figure1.** A statistical framework for estimating absolute risk differences between cerebral embolic protection (CEP) and control at 30 days.

6 trials of CEP vs. control

(3,921 individuals)

**Relative risk (95% CI)**

**for clinical outcomes**

**Baseline risk of any stroke and all-cause mortality from TAVI vs. SAVI trials at 30 days**

| **High or prohibitive risk** |
| --- |
| PARTNER 1A |
| PARTNER 1B |
| US CoreValve |
| **Intermediate surgical risk** |
| PARTNER 2 |
| SURTAVI |
| **Low surgical risk** |
| NOTION |
| PARTNER 3 |
| Evolut Low risk |

**Estimation of absolute risk difference between CEP and control per 1000 at 30 days** using MAGICapp (https://magicevidence.org/)


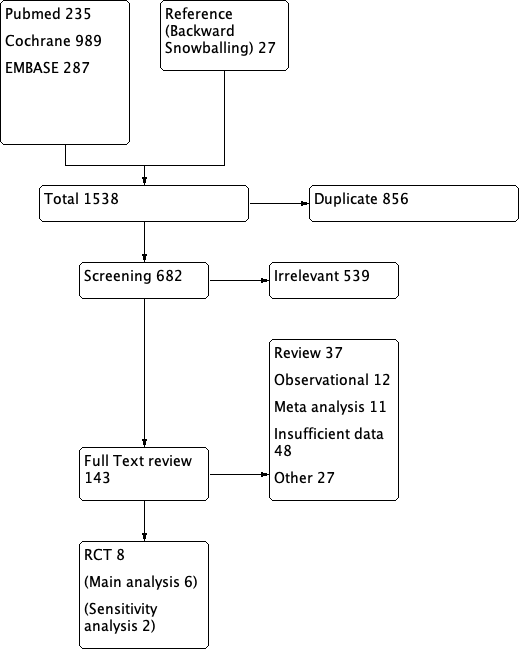
**Online Figure 2.** Study selection process.

**Online Figure 3.** Sensitivity analysis after adding CLEAN TAVI and EMBOLI-X trials.

**
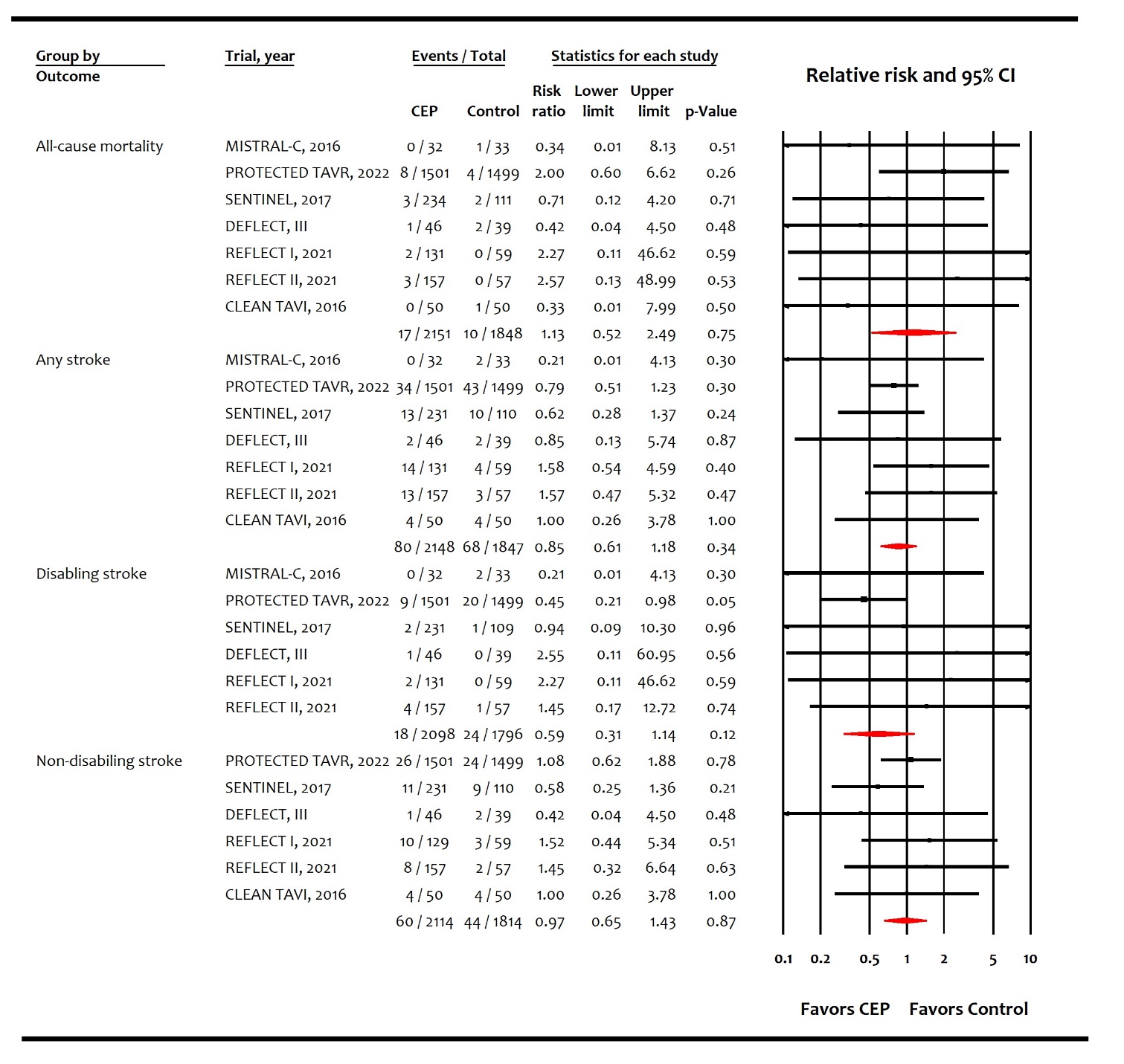
**
